# Parathyroid adenoma with rare severe pathological osteolytic lesion: a case report and literature review

**DOI:** 10.3389/fonc.2023.1218863

**Published:** 2023-08-03

**Authors:** Jia Chen, Gusheng Tang, Ye Peng, Hui Cheng

**Affiliations:** ^1^ Department of Hematology, Naval Military Medical University, First Affiliated Hospital, Shanghai, China; ^2^ Department of Nuclear Medicine, Naval Military Medical University, First Affiliated Hospital, Shanghai, China

**Keywords:** parathyroid adenoma, parathyroid hormone, serum calcium, pathological osteolytic lesions, parathyroidectomy

## Abstract

Parathyroid adenomas are benign proliferative disorders of parathyroid glands. Patients typically exhibit hyperparathyroidism and elevated serum calcium levels due to elevated levels of parathyroid hormone (PTH). We report a newly diagnosed case of a rare pathological osteolytic lesion. Radiological evaluation revealed multiple bony lesions in multiple parts of the pelvis, vertebral body, and spinous process, suggesting hematological neoplasms or bone marrow metastatic carcinoma. The morphology revealed many abnormal cells in the bone marrow smear. Furthermore, serum calcium and PTH levels were significantly increased compared to normal levels. Doppler color ultrasound showed a thyroid mass (left), suspected parathyroid adenoma, thyroid, and isthmus nodular goiter (right). The patient underwent bilateral neck exploration with parathyroidectomy, and serum calcium and PTH levels significantly decreased on the second day after surgery and had a surgical cure.

## Introduction

Parathyroid adenoma is a type of parathyroid proliferative disease, including parathyroid hyperplasia, parathyroid adenoma, and parathyroid cancer. Eighty to 85% of patients with parathyroid adenomas typically exhibit hyperparathyroidism (1). Hyperparathyroidism has the potential to be aggressive and destructive (2). Bone involvement in hyperparathyroidism can manifest as subperiosteal resorption, systemic demineralization, or focal lytic lesions (3). However, only a few patients experience severe pathological osteolytic lesions. Herein, we report a unique case of pathological osteolytic lesions and the pretreatment diagnostic challenges faced in the differential diagnosis of a potential hematological neoplasm or bone marrow metastatic carcinoma.

## Case report

**Figure 1:**
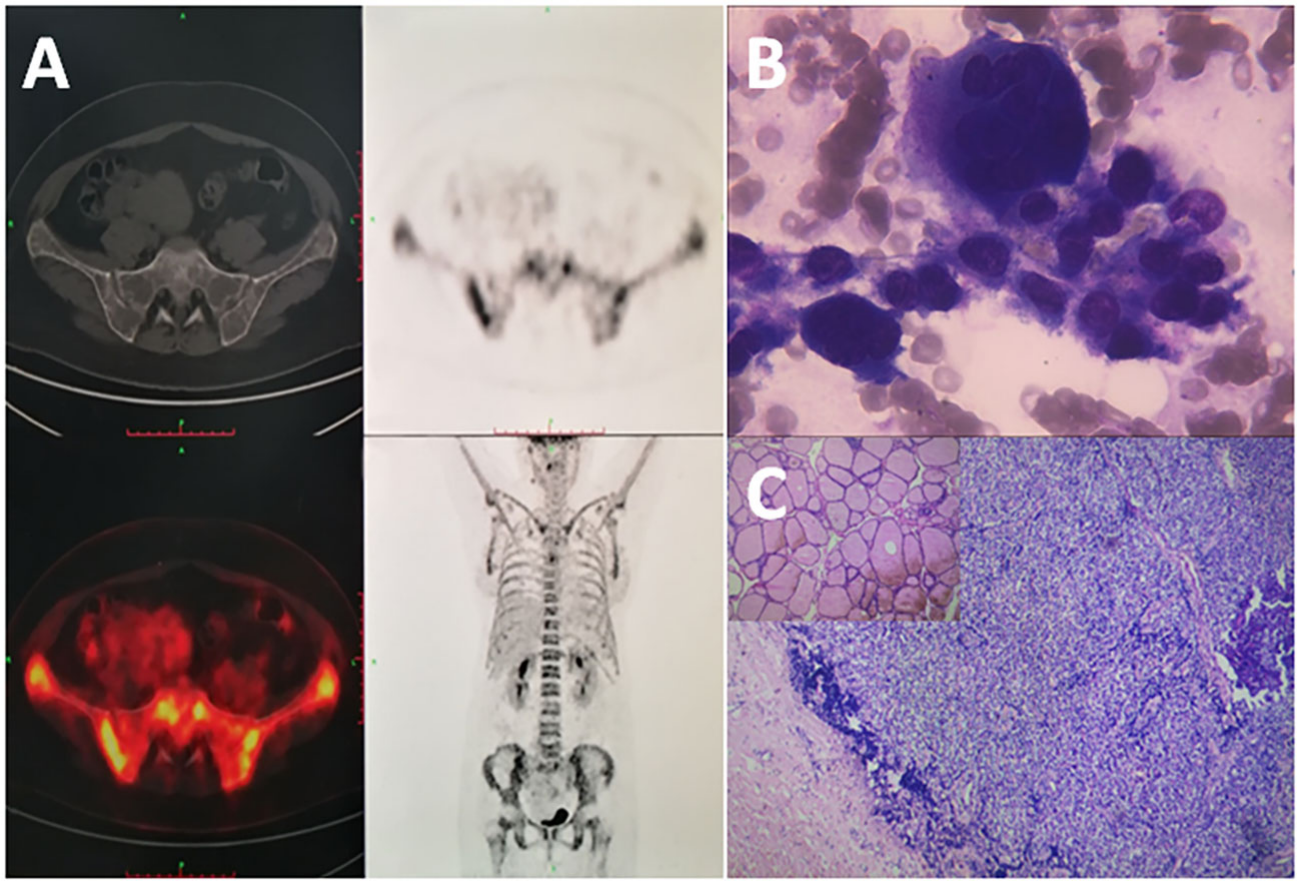
**(A)** Multiple low-density shadows and hypermetabolism in multiple parts of the pelvis with no significant imaging in the parathyroid gland on PET/CT examination. **(B)** Nuclear cells on bone marrow smear: Focal or fused distribution with deep staining of cytoplasm and nucleus (×1,000, Wright-Giemsa). **(C)** Thick collagen fiber capsule of the lesion (×100, hematoxylin–eosin), focal distribution of water-like transparent cells, with few stroma (×400, hematoxylin–eosin stain).

**Figure 2:**
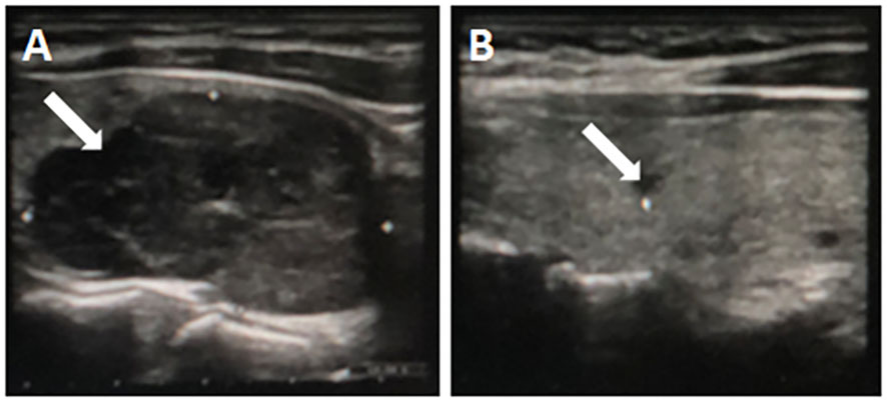
A large homogeneous mass (4.1 × 2.6 cm) with distinguishable boundaries in the lower pole of the left thyroid gland **(A)**, a hypoechoic nodule (0.4 × 0.2 cm) with unclear boundaries in the middle of the right thyroid gland **(B)**.

**Figure 3:**
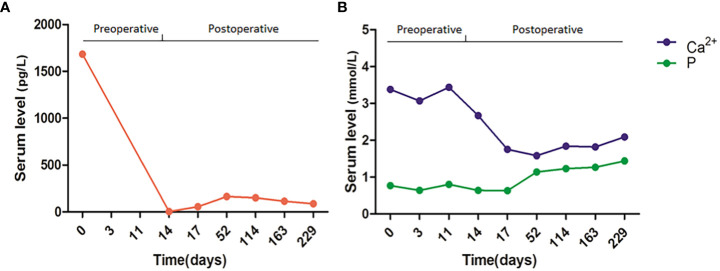
Dynamic monitoring of PTH **(A)** and serum calcium and phosphorus **(B)** levels preoperatively and postoperatively.

A 42-year-old woman complained of general weakness for one week and had a history of fracture. Physical examination revealed no other special findings, except for bone pain. Complete Blood Count displayed small cell hypopigmentation anemia, and serum iron 3.60 μmol/L (normal, 9–27 μmol/L), unsaturated iron binding force 65.63 μmol/L (normal, 25–52 μmol/L). Accordingly, this patient was supposed to be diagnosed as iron deficiency anemia (IDA). However, computed tomography (CT) showed multiple low-density shadows in the L3 vertebral body, sacrum, pubis, ischium, and both femurs. Positron emission tomography-computed tomography (PET-CT) also showed elevated metabolism in the pelvis, suspected hematological neoplasms, and bone marrow metastatic carcinoma (**Figure 1A**). Immunofixation electrophoresis (IgG, IgA, IgM, κ, and λ) was negative. Many abnormal cells that were difficult to distinguish from multiple myeloma and metastatic carcinoma were found in the bone marrow smear (**Figure 1B**). Doppler color ultrasound showed a thyroid mass (left, 4.1 × 2.6 cm) (**Figure 2A**) and suspected parathyroid adenoma, thyroid, and isthmus nodular goiter (right, 0.4 × 0.2 cm) (**Figure 2B**). Blood biochemical index indicated serum calcium 3.38 mmol/L (normal, 2.11–2.52 mmol/L), serum phosphorus 0.77 mmol/L (normal, 0.82–1.62 mmol/L) and PTH 1,684 pg/L (normal, 15–65 pg/L). Therefore, parathyroid adenoma (left), thyroid, and isthmus (right) resections were performed under general anesthesia. Histopathological examination confirmed the diagnosis of parathyroid adenoma (**Figure 1C**). Pathologically, the left parathyroid gland area had 4 × 3.6 × 2.8 cm size nodular mass, section grayish-yellow, solid, soft, with an intact surface envelope. Right thyroid and isthmus with 5.7 × 5.5 × 1.1 cm size gray red tissue, a nodule with a diameter of 0.2 cm can be seen on the section at a distance of 0.2 cm from the capsule on the section, section gray white, solid, hard, with unclear boundaries. The patient recovered well postoperatively, serum calcium (2.67 mmol/L), serum phosphorus (0.64 mmol/L) and PTH (4.49 pg/L) also significantly decreased on second day after surgery (**Figure 3**).

## Discussion

The parathyroid gland produces parathyroid hormone (PTH), which plays a key role in regulating the calcium balance in the body (4). Parathyroid adenoma is a type of parathyroid hyperplastic disease, which is characterized by primary hyperparathyroidism with elevated serum calcium and PTH levels. Patients with hyperparathyroidism present non-specific symptoms, such as fatigue, pain, and weakness (1).

This patient started with fatigue, and CT imaging showed low-density shadows of multiple parts of the body. MRI showed bone destruction of the L3 vertebral body and spinous process, and PET/CT fusion image showed bone destruction of the bilateral ilium with abnormal metabolism. Osteolytic lesions are common imaging changes in multiple myeloma and bone metastatic carcinomas (5, 6). However, the patient who underwent immunofixation electrophoresis yielded negative results. Many abnormal cells that were difficult to distinguish from osteoclasts were found in bone marrow smears. These cells were relatively large and different in size, and could be seen as polynuclear, the nuclei were mostly quasi-circular, the nuclear chromatin was relatively gathered, the cytoplasm was rich, dark blue, and the fusion between cells was observed, and hematological neoplasms or metastatic carcinoma were suspected.

However, Doppler color ultrasound revealed a thyroid mass, thyroid, and isthmus nodular goiter, while serum calcium and PTH levels were significantly increased. Based on these results, the patient was clinically diagnosed with parathyroid adenoma. Therefore, the parathyroid adenoma, thyroid, and isthmus goiter were removed under general anesthesia. Based on the histopathology and clinical context, the tumor was identified as a parathyroid adenoma. The serum calcium and PTH levels also decreased significantly after parathyroidectomy.

Parathyroid adenoma is a benign tumor of the parathyroid gland that involves a single gland and is usually the main cause of primary hyperparathyroidism (80%), followed by parathyroid hyperplasia (15%), ectopic parathyroid adenoma (4%), and multiple parathyroid adenomas (1–2%) (7). In the differential diagnosis, it is necessary to distinguish it from multiple endocrine neoplasias because polyglandular and syndromic disorders associated with primary hyperparathyroidism are more likely (8). Nevertheless, this patient did not have any symptoms suggestive of syndromic involvement such as galactorrhea, headache, visual deficit, neuroglycemic symptoms, thyroid nodules or goiter, adrenergic phase, hypertension, or jaw tumor.

Parathyroid adenomas mainly occur in female aged 40–80 years (9, 10). Increased PTH serum levels are a common cause of hypercalciuria, which can manifest as recurrent kidney stones (11). However, in our patient, Doppler color ultrasound revealed no abnormalities or stones in the urinary system. Iwen et al. reported that the sestamibi scan has 68%–72% sensitivity and 99% specificity for detecting a parathyroid adenoma and can significantly improve the accuracy of diagnosis and the success of surgery (12). Thus, according to the meta-analysis, there were no significant differences between ultrasonography and parathyroid scintigraphy with 99mTc-MIBI in terms of sensitivity and specificity. There was overlap in the 99% confidence interval (13).

Currently, parathyroidectomy is the best choice for hyperparathyroidism to reduce the long-term destructive effect on bone and the negative effect of high serum calcium levels (14). Prevention of postoperative “hungry bone syndrome” is also important. The clinical symptoms caused by the rapid decline of high circulating levels of PTH after parathyroidectomy include bone deformation, fracture, and hypocalcemia (<2.1 mmol/L), which is called “Hungry Bone Syndrome” (15). Therefore, the patient was administered intravenous calcium repletion on the second postoperative day, followed by high-dose oral calcium and calcitriol supplementation and monitoring of serum calcium and PTH levels. At follow-up, the patient recovered well, and the serum calcium and PTH levels were within the normal range.

## Conclusion

This is an extremely rare and easily misdiagnosed severe osteolytic lesion caused by parathyroid adenoma. This report contributes to improving the awareness of clinicians and pathologists that the clinical, radiologic, and cytomorphological suspicion of hematological neoplasms or metastatic carcinoma may also be benign tumors. Clinicians and pathologists must keep in mind that parathyroid adenomas can cause osteolytic lesions and increase the number of osteoclasts, especially in hyperparathyroidism.

## Data availability statement

The original contributions presented in the study are included in the article/supplementary material. Further inquiries can be directed to the corresponding author.

## Ethics statement

Written informed consent was obtained from the participant/patient(s) for the publication of this case report.

## Author contributions

JC collected clinical data. GT and YP diagnosed and followed up the patient. HC reviewed the literature and prepared the initial draft of the manuscript. All authors contributed to the article and approved the submitted version.
